# Experimental data for extrusion processing and tensile properties of poly(hydroxybutyrate-co-hydroxyvalerate) (PHBV) polymer and wood fibre reinforced PHBV biocomposites

**DOI:** 10.1016/j.dib.2018.12.084

**Published:** 2018-12-29

**Authors:** Luigi-Jules Vandi, Clement Matthew Chan, Alan Werker, Des Richardson, Bronwyn Laycock, Steven Pratt

**Affiliations:** a School of Chemical Engineering, University of Queensland, St. Lucia, Queensland, Australia; b Promiko AB, Lomma, Sweden; c Norske Skog Paper Mills (Australia) Ltd, Boyer, Tasmania, Australia

## Abstract

This article features a large database on different extrusion processing conditions and the resulting tensile properties of poly(hydroxybutyrate-*co*-hydroxyvalerate) (PHBV) and wood fibre reinforced biocomposites. The data presented here corresponds to a comprehensive design of experiments conducted separately for both neat PHBV polymer and wood–PHBV composites, in which the effects of temperature profile, screw speed, feeding rate, feeding method, screw configuration, and wood contents (wood–PHBV composites only) of 10, 20, 30, and 40 wt% wood content were examined. For each processing condition, 5 specimens were tested under uniaxial tensile loading. Here we provide the complete set of extrusion parameters, including the observed screw torque, residence time and material output. Individual stress–strain curves for each specimens are provided, along with their calculated elastic modulus, strength, and strain at maximum load. The data is also provided as support material for the research article: “Extrusion of wood fibre reinforced Poly(hydroxybutyrate-co-hydroxyvalerate) (PHBV) biocomposites: statistical analysis of the effect of processing conditions on mechanical performance” (Vandi et al., 2018).

**Specifications table**

**Table:** 

Subject area	Materials Science
More specific subject area	*Biopolymers and Biocomposites*
Type of data	*Table, graph*
How data was acquired	*Uniaxial tensile testing conducted according to ASTM D638 (Type V specimens), using an electromechanical Instron model 5584.*
Data format	*Raw, analyzed*
Experimental factors	*Extruded samples were conditioned at a controlled temperature of 25* °*C and humidity of 40–60% for at least two weeks, prior to laser cutting into ASTM Type V dog bone specimens, and testing.*
Experimental features	*Large set of tensile properties for PHBV and wood–PHBV composite extruded through different processing conditions.*
Data source location	http://dx.doi.org/10.17632/9fzzyp63hp.1
Data accessibility	*Data is presented in this article and in Mendeley Data, v1.*
Related research article	*Vandi, L.-J., C.M. Chan, A. Werker, D. Richardson, B. Laycock, and S. Pratt, Extrusion of wood fibre reinforced poly(hydroxybutyrate-co-hydroxyvalerate) (PHBV) biocomposites: Statistical analysis of the effect of processing conditions on mechanical performance. Polymer Degradation and Stability, 2018*. https://doi.org/10.1016/j.polymdegradstab.2018.10.015

**Value of the data**

• The database presented here can be used to find optimal processing parameters for neat PHBV polymer and wood–PHBV composites of different wood contents.
• The data is useful to understand the effect and contribution of individual extrusion parameters on the mechanical properties of PHBV and wood–PHBV materials.
• The calculated tensile properties can serve as a benchmark for the performance of PHBV and wood–PHBV composites, in comparison to other PHA copolymers.
• The mean and standard deviation values of mechanical properties can be used to determine design allowables for a component design.

## 1 Data

This article features raw stress–strain tensile data with the calculated tensile modulus, strength and strain at maximum load for more than 300 specimens corresponding to PHBV and wood–PHBV materials extruded under different conditions. For each sample the extrusion processing parameters are reported and include: feeding method, feeding rate, fibre ratio, screw configuration, screw speed, temperature profile, observed screw torque, residence time, and material output. Finally, we include a summary table with processed data for neat PHBV and wood–PHBV material separately. The complete data can be found in the file ‘Data in Brief - wood–PHBV Composites.zip’ available in the Mendeley data repository under the following identifier DOI: https://doi.org/10.17632/9fzzyp63hp.1.

## 2 Experimental design, materials and methods

### 2.1 Experimental design

A design of Experiment (DoE) through a 32-run Taguchi orthogonal array was applied for this investigation. 1 factor at 2 levels and 4 factors at 4 levels (L32 2ss1 4ss4) were used to define experimental runs for wood–PHBV composites. The full test matrix, including manufacturing parameters and the values investigated is shown in Table 1. A second study with neat PHBV was conducted using identical factors and levels to the first DoE, excluding: fibre/matrix combination, and fibre ratio, since no fibres were introduced into the extruder. This led to a 16-run array of 3 factors at 4 levels (L16 4ss3), where each run is a replica, without any wood fibres, of the 32-run wood–PHBV experimental design from Table 1. The order of testing experiment factors was randomly assigned, to reduce the potential influence of a time dependent bias. The sample labels listed in the file ‘*Full Design of Experiments*’ is the order in which samples were run through the extruder. The labelling nomenclature was 1A to 32A for neat PHBV material, and 1B to 32B for wood–PHBV composites. Within each sample, individual dog bone specimens were labelled 1–5. A color code is used in the file ‘*Full Design of Experiments*’ to help identify the four different levels of varying extrusion parameters. The recorded output values include screw torque (N m), residence time (s), material output (g/min) during extrusion (shown in Table 1), and tensile stress at maximum load (MPa), tensile strain at maximum load (%), and tensile modulus (GPa) during mechanical testing, shown in the file ‘*Full Design of Experiments*’.

**Table 1:** Full test matrix showing parameters investigated for wood–PHBV composites.

Feeding style	Fibre ratio (%wt)	Screw configuration	Screw speed (rpm)	Feeding rate (cm^3^/min)	Temp (°C)	Screw torque (N m)	Residence time (s)	Output (g/min)
Disintegrated	10	Standard	50	10	170	2.75	90	2.3
Disintegrated	10	Standard	100	40	180	4.70	57	8.0
Disintegrated	10	Standard	150	Full barrel	190	5.25	34	13.8
Disintegrated	10	Standard	200	Full barrel	210	4.65	21	16.2
Disintegrated	20	Standard	50	40	170	5.00	110	–
Disintegrated	20	Standard	100	10	180	2.10	50	1.7
Disintegrated	20	Standard	150	Full barrel	190	4.90	35	9.5
Disintegrated	20	Standard	200	Full barrel	210	5.15	29	12.1
Disintegrated	30	Standard	100	Full barrel	170	5.85	75	7.2
Disintegrated	30	Standard	50	Full barrel	180	4.85	107	3.9
Disintegrated	30	Standard	200	10	190	1.80	35	1.6
Disintegrated	30	Standard	150	40	210	2.80	24	3.3
Disintegrated	40	Standard	100	Full barrel	170	5.35	65	5.4
Disintegrated	40	Standard	50	Full barrel	180	3.95	140	4.9
Disintegrated	40	Standard	200	40	190	3.75	20	4.0
Disintegrated	40	Standard	150	10	210	2.25	37	1.4
Light mixing	10	Standard	200	10	170	1.40	80	–
Light mixing	10	Standard	150	40	180	3.55	45	8.7
Light mixing	10	Standard	100	Full barrel	190	5.25	69	15.9
Light mixing	10	Standard	50	Full barrel	210	4.65	115	8.6
Light mixing	20	Standard	200	40	170	3.90	47	8.6
Light mixing	20	Standard	150	10	180	1.75	120	–
Light mixing	20	Standard	100	Full barrel	190	6.55	90	11.4
Light mixing	20	Standard	50	Full barrel	210	4.00	72	6.7
Light mixing	30	Standard	150	Full barrel	170	5.95	56	–
Light mixing	30	Standard	200	Full barrel	180	6.20	34	15.3
Light mixing	30	Standard	50	10	190	3.10	160	1.4
Light mixing	30	Standard	100	40	210	–	80	–
Light mixing	40	Standard	150	Full barrel	170	6.35	78	8.7
Light mixing	40	Standard	200	Full barrel	180	8.10	28	12.4
Light mixing	40	Standard	50	40	190	5.00	180	4.2
Light mixing	40	Standard	100	10	210	2.85	89	1.1
Light mixing	20	Aggressive	100	Full barrel	190	9.05	110	9.3
Light mixing	30	Aggressive	200	Full barrel	180	11.00	–	14.4

### 2.2 Materials

The type of polyhydroxyalkanoate (PHA) polymer used was Poly(3-hydroxybutyrate-*co*-3-hydroxyvalerate) (PHBV) with 1 mol% 3-(hydroxyvalerate) (HV) content. This was purchased from TianAn Biopolymer, China, in powder form under the trade name of ENMAT Y1000 [2]. The density is 1.25 g/cm^3^, and a reported melt flow index (MFI) of 5.2 g/10 min at 180 °C, using a weight of 2.16 kg, according to ISO 1133 [3]. The melting temperature of ENMAT Y1000 was found to be 171 °C [1], as measured by Differential Scanning Calorimetry (DSC) in accordance with ASTM D3418-12.

Thermomechanical pulp (TMP) wood fibres from radiata pine wood, were supplied as unbleached by Norske Skog paper mill, Boyer, Tasmania. A contoured weighted average length L(c) of 2.36 mm, fibre width (W) of 33.6 μm, and a fibre wall thickness (or cell wall thickness, CWT) of 5.0 μm were obtained through characterisation with a FiberLab^™^ V3.0 analyser. Further characterisation of the fibres and density measurements can be found in [1].

### 2.3 Extrusion processing

A EuroLab 16 XL co-rotating twin screw extruder (ThermoFisher Scientific Inc, Waltham, USA) with a diameter of 16 mm and a length-to-diameter ratio of 40:1 was used for this work. Residence time was measured for each extrusion run by introducing ~1 g of red tracer and monitoring the time until it was first observed at the die exit. Temperature profiles at 170, 180, 190, and 210 °C were investigated in this study. The individual barrel temperature for these temperature profiles is described in [1].

The screw configurations chosen for this study is shown in Table 2. At the end of the design of experiments a separate study with a more aggressive mixing region in the screw design was conducted by repeating two runs (at 20 wt% and 30 wt% fibre content), to investigate its effect on fibre dispersion through mechanical performance. These are labelled 06C, 16C for neat PHBV and 06D, 16D for wood–PHBV composites. The screw configuration used in each extrusion run is clearly indicated in Column E (Screw Configuration) of the file ‘*Full Design of Experiments*’ and referred to as ‘Standard Mixing zone’ and ‘Aggressive mixing zone’.

**Table 2:** Screw profile and assembly for ‘standard’ and ‘aggressive’ configurations.

### 2.4 Tensile testing

**Fig. 1:**
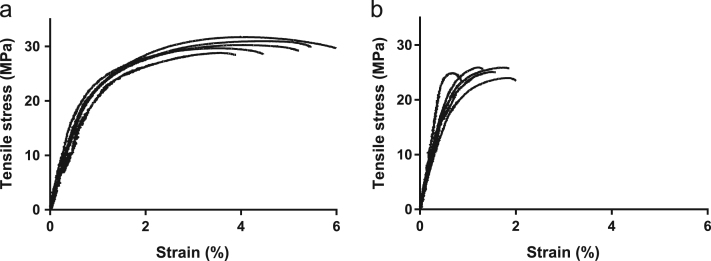
Typical tensile curves observed for (a) neat PHBV, and (b) wood–PHBV (30 wt%) specimens.

Specimens to be tested were conditioned at a controlled temperature of 25 °C and humidity of 40–60%, during two weeks prior to testing. This allows sufficient time for secondary crystallisation of PHBV [1]. Standard procedures according to ASTM D638 (Type V specimens), were followed during tensile testing using an electromechanical Instron model 5584 (Instron Pty Ltd, USA) with a 1 kN load cell. Specimens were tested at constant cross-head displacement rate of 1 mm/min and clamped using pneumatic grips. The strain value across the narrow region of the specimen was monitored using a video-extensometer. A total of five specimens were tested for each extrusion run. Typical tensile stress versus strain curves observed for neat PHBV and wood–PHBV composites are shown in Fig. 1. The raw data files containing stress–strain values for each individual tested specimen are located in two separate folders ‘*Raw Data for mechanical testing – Samples A*’ and ‘*Raw Data for mechanical testing – Samples B*’ for neat PHBV material and wood–PHBV composites respectively. More specifically the raw data files include values for time (s), extension (mm), load (N), video axial strain (%) and tensile strength (MPa), as well as each specimen dimensions (width, thickness and gauge length) and the test date. In this work, tensile strength was calculated as the tensile stress at maximum load (in MPa). Tensile modulus (in GPa) was calculated by the linear trend from fitting between 0% and 2.0% strain, while strain (in % video axial strain) was measured at the point of maximum load. A summary of all the processed data for neat PHBV material and wood–PHBV composites is available in root folder in the files ‘*Summary of mechanical testing – Samples A*’ and ‘*Summary of mechanical testing – Samples B*’ for neat PHBV and wood–PHBV composites respectively.
